# ‘When the bedbugs come, that’s another problem’: exploring the lived experiences of bedbug infestations among low-income older adults and service providers who support them

**DOI:** 10.1177/17579139221118777

**Published:** 2022-09-20

**Authors:** CL Sheppard, B Roche, A Austen, SL Hitzig

**Affiliations:** St. John’s Rehab Research Program, Sunnybrook Research Institute, Toronto, ON, Canada; Wellesley Institute, Toronto, ON, Canada; Seniors Services and Long-Term Care, City of Toronto, Toronto, ON, Canada; St. John’s Rehab Research Program, Sunnybrook Research Insitute, 285 Cummer Avenue, Toronto, ON M2M 2G1, Canada

**Keywords:** bedbugs, pest control, integrated pest management, older people, housing, health services

## Abstract

**Aims:**

Older adults in low-income housing communities are more vulnerable to bedbug infestations. Prior research, however, has predominately focused on the effectiveness of integrated pest-management strategies, with little attention given to the lived experiences of tenants struggling with infestations. We used a qualitative approach to explore what it is like to live with and treat bedbug infestations from the perspectives of low-income older adults and service providers.

**Methods:**

Participants included low-income older adults (*n* = 58) and service providers (*n* = 58) who offer supports directly in the buildings. Semi-structured qualitative interviews and focus groups were used to explore the challenges of preparing and treating units for bedbugs, and examine how bedbugs impact access to support services.

**Results:**

Bedbugs were a widespread issue, and underlying physical, mental, social, and financial challenges made it difficult for older tenants to prepare their units and access treatment. Tenants also faced bedbug stigma from community services, as many were unwilling to provide services in infested units. Although some service providers utilized strategies to minimize exposure, many were concerned these strategies created additional stigma.

**Conclusion:**

Our findings highlight an urgent need to increase public health funding to support older adults with the costs of bedbug elimination and to enhance pest-management strategies through partnerships with health and social service agencies to improve outcomes for older adults.

## Introduction

Over the past 20 years, there has been a global resurgence of bedbugs.^1^ Bedbugs are found in dark places, such as mattresses, bed frames, furniture, and baseboards.^2^ They emerge at night for blood meals from a sleeping human host; with continual feeding access, bedbugs multiply exponentially in a few short months, leading to large infestations.^3^ Bedbugs are prevalent in low-income communities,^3–5^ where ineffective pest management has been linked to insufficient financial resources, low awareness among residents, and an inability to prepare units for treatment.^6–10^ Control of bedbugs requires an integrated pest-management strategy that combines chemical (i.e. insecticide) and non-chemical (e.g. steaming, encasement, vacuuming, de-cluttering) elimination strategies with early detection, education, and outreach.^7^

Bedbug infestations represent a significant public health concern.^11^ In addition to the economic impact,^12^ the health consequences are tremendous: the bites lead to itchy and painful lesions and rashes,^5^ and infestations cause sleep disturbances,^3^ psychological distress, and stigma.^13^ The detrimental impacts of bedbugs may be more severe for older adults,^13^ who are least likely to be aware of and report infestations.^14^ For instance, Wang et al.^15^ found that awareness of bedbugs among low-income older adults was low, yet 45% of units were infested. When infestations were identified, older tenants struggled with treatment compliance due to limited financial resources and physical disabilities.^9^ Older residents with unmanaged pest infestations may also be at risk of losing access to in-home health and social services, as service providers may be hesitant to go into infested homes.^16^

Much of the research to date has focused predominately on understanding the scope and clinical relevance of bedbug infestations and evaluating integrated pest-management strategies.^3,7^ There is a dearth of research on the lived experiences of those with chronic infestations. For example, a recent scoping review on the mental health impacts of bedbugs found that only 5 of 51 reviewed articles presented original research.^13^ There is also a lack of qualitative evidence, which limits our understanding of what it is like to live with and treat an infestation from the perspective of tenants. Given that low-income older adults may be more vulnerable to infestations,^9,13,15,17^ it is critical to understand the unique challenges that they face living with bedbugs. This study used a qualitative approach with low-income older adults, as well as with service providers who support them, to explore: (1) the challenges older tenants face preparing their units and receiving treatment and (2) how bedbug infestations impact their access to in-home health and social services.

## Methods

This article is based on data from a larger qualitative study aimed at understanding the lived experiences of older adults residing in social housing in Toronto, Canada.^18^ Pest control emerged as a pervasive issue negatively impacting quality of life for tenants. Therefore, this article explores the challenges of managing bedbug infestations from the perspectives of older adults and service providers who support them.

### Study context

This study was conducted in Toronto, which was ranked as Canada’s top bedbug city in 2019 and 2020.^19^ We worked with a social housing provider that is home to over 35,000 older adults (aged 59 years or older). The housing provider recognizes that effective pest management is essential for building healthy communities; however, pest infestations have been on the rise. For example, recent news articles highlight how mail services were halted in a seniors’ building due to an ongoing bedbug infestation in the mailroom. Furthermore, in 2020 the housing provider received over 50,000 service requests for pest infestations, predominately bedbugs. To manage infestations, the housing provider uses an integrated pest-management approach that incorporates community engagement, education, proper preparation for treatment, and responsible use of pesticides.

### Sample

Older adults (*n* = 58) were recruited through flyers placed in common areas of their building (e.g. lobby). Interested tenants contacted the researcher to schedule an interview or sign up for a pre-scheduled focus group. Characteristics of participating tenants are shown in Table 1.

**Table 1:** Tenant characteristics

Characteristics	Mean value ± standard deviation (range)	% (*n*)
Age	70.4 ± 8.4 years (57–92 years)	
Gender (male)		50% (*n* = 29)
Live alone		75% (*n* = 44)
Length of tenancy	9.1 ± 8.4 years (3 months–38 years)	

Service providers (*n* = 58) were recruited through flyers distributed to various health and social service agencies that operated in the buildings. Interested participants contacted the researcher to schedule an interview or sign up for a pre-scheduled focus group. Service providers represented frontline and management roles from a variety of sectors, including social work, nursing, housing, psychiatry, supportive housing, care coordination, and community support services (CSS).

### Data collection

Interviews were conducted by telephone or in-person at an agreed upon location. In addition, six focus groups (two with tenants and four with service providers) were carried out. Tenants had the option to complete their interview in English (*n* = 41), Chinese (*n* = 14), or Tamil (*n* = 3). Data were collected between November 2019 and February 2020. All sessions were facilitated by a trained interviewer and lasted approximately 1 h. The discussion explored several topics related to tenancy management and access to services. Sample interview questions related to pest management are provided in Table 2. All sessions were audio-recorded and transcribed verbatim. Non-English transcripts were professionally translated and reviewed by the interviewer for accuracy. All transcripts were uploaded into NVivo 12 for analysis.

**Table 2:** Sample interview questions

Tenant interview questions: • When you think about what makes a ‘home’, what three words come to mind? • What do you like most about your home? • What helps you to maintain your apartment (e.g. keep it free of pests)? • What makes it challenging for you to keep your apartment in good shape?
Service provider interview questions: • When you think about what makes a ‘home’ for older tenants, what three words come to mind? • What are the main challenges older adults face maintaining their unit? • What factors influence how successful tenants are at maintaining their unit? • When tenants have challenges with their unit condition (e.g. pests), how do you work with housing staff/service providers to support them?

Ethics approval was granted from the Sunnybrook Health Sciences Centre, and informed consent was obtained from all participants at the beginning of the study. Tenants received a CAD$25 gift card for their participation while service providers received refreshments or a CAD$10 gift card.

### Analytic approach

Our research team utilised a qualitative descriptive approach^20^ to elicit a rich description of bedbug management from the perspective of older adult tenants and service providers. This approach is widely used in health research^21,22^ due to the emphasis on learning from lived experiences and using that knowledge to influence policy and practice.^20^ Following the principles outlined by Braun and Clark^23^ and Saldana,^24^ transcripts were read and re-read, and line-by-line coding was carried out using the method of constant comparison. Rigour was established through a combination of techniques including double-coding, audit trails, memoing, and team meetings.

Following our analysis, a composite narrative was created to draw together the shared experiences of tenants managing a bedbug infestation. Composite narratives draw on data from several interviews to create a single story that reflects a common understanding,^25^ and they give voices to groups with unique lived experiences.^26–28^ The choice for a composite was also driven by the personal and compelling stories shared by tenants. Following the steps described by Willis,^25^ the narrative was created out of coded data emerging from tenants who had lived through persistent bedbug infestations that spanned many treatment cycles. The creation of the narrative was iterative, involving repeated cycles of reviewing the data and writing the narrative. The final narrative synthesizes the lived experience of tenants and conveys the richness of their stories through verbatim quotes.

## Results

Bedbugs were a ‘paramount issue [. . .] regardless of what building or what part of the city’ (SP30, Care Coordinator). They were a constant source of fear, and tenants felt that no matter how hard they worked, the bedbugs always came back. Infestations had reportedly increased over the past two decades, with one geriatric psychiatrist describing how ‘this is something I never thought I would talk about, I literally never dealt with it in the 80’s and 90’s [. . .] and now it’s become a nightmare’ (SP14, Geriatric Psychiatrist). In fact, infestations had become so widespread that this participant felt the housing provider was ‘fighting a losing battle – they’re like a finger in a dike but they don’t have enough fingers’.

Many tenants had repeated experiences with bedbugs; the composite narrative shown in Box 1 reflects this experience. The remainder of our results discuss the challenges of preparing and treating units for bedbugs, including the reasons that make unit preparation challenging for older tenants, the difficulties coordinating treatment, and the impact of bedbugs on accessing in-home health and social services.

**Box 1:** Rose.

Rose is 70 years old. For the past 5 years, she has lived alone in her bachelor apartment. Unfortunately, Rose has bedbugs. She has had them six or seven times since she moved in, and each time it destroys her life. She is not sure where they came from this time – she thought she had been so careful to avoid her neighbour who she knew had refused bedbug treatment. Rose puts out a powder she got from the hardware store to try and stop them, but the infestation continued to grow: ‘[The bedbugs] basically ended up [in] my couch, behind my bookcase, behind my wall unit. They were in my bedroom, they were actually in my bed [. . .] and literally when I would lie down to go to bed, I could feel them moving [on me]’.
Rose was afraid to tell her housing provider – she is worried they will make her throw out her things or that they might ask her to leave because she keeps having issues. After a few weeks, she finds the courage to report the infestation, and housing staff tell her that a pest control company would be coming to spray her apartment next week and that she needed to prepare her unit. Even though Rose is ‘one of the more fit seniors in the building’, she knows from experience that ‘the prep is extremely hard’ and she cannot do it on her own. Even though it’s not her job, her social worker put on a ‘hazmat suit’ and helped Rose throw out her bed and her couch, which she was told were too infested to clean. Since Rose cannot afford to buy new furniture, her social worker is going to make a referral to the local furniture bank to replace the items that were thrown out, but until then, she will have to ‘sleep on the floor’.
Throwing out her belongings makes Rose upset, but she knows it’s her only option. ‘I don’t like losing my stuff, but I do. And the only way to get rid of [the bed bugs] is to get rid of all the stuff you have’. Together, they also packed away all of Rose’s clothes, and put the rest of her belongings into freezer bags, including her books and pictures, so that the bugs won’t get them. ‘Everything I had in my apartment was all in plastic bags in the middle of my floor or on my kitchen table. I cannot move in my apartment, [. . .], I’ve got maybe [two or three feet] of space that I can maneuver in. [. . .] It looks like a tsunami’.
Since her apartment is so small, Rose ‘filled up [her] bathtub [with bags], so every time [she] wanted to take a shower, [she] had to empty [it] out’. Rose is worried about how long she’ll have to live this way; she knows it will be at least 6 weeks before she can unpack her things, but it could be longer. With all her stuff in bags, she no longer feels safe or at home in her apartment. She also doesn’t want her friends to find out – what if they start avoiding her now?
Rose does not trust her housing provider to get rid of the bedbugs. The contractors rarely show up, and when they do, they ‘are in and out in five minutes’. Tomorrow, she is going to go out and buy a steamer so that she can ‘steam the bejeebers out of everything’ like the baseboards and her chairs. Rose hopes that this will help her have more control in her life and stop living out of bags.

### Preparing the unit – ‘If things don’t get prepped properly, you’ll never get rid of the bedbugs, but you can’t expect a senior to be able to prep their unit’

Service providers and tenants stressed the importance of the pre-treatment preparations, emphasizing that the unit ‘has to be prepared in a flawless way’ (SP13, Supportive Housing Manager) or else the bedbugs return; however, there was a widespread understanding that older adults face obstacles preparing their unit. As one service provider described, ‘they might have the wherewithal to prep but they don’t have the physical ability. Or they might have mental health [challenges] and not understand how to prep or comply with the requirements’ (SP9, CSS Manager). These physical and mental health challenges were exacerbated by a lack of financial and social resources that would usually be drawn upon in the absence of accessible prep services (see Table 3).

**Table 3:** Challenges faced preparing units for treatment

Challenge	Supporting quote
Lack of education and awareness	‘A lot of times people won’t say anything if they see it’s a problem. Others don’t see the problem. ‘Oh, it’s couple of bugs’ meanwhile they are everywhere. And so, that’s challenging, as well as them understanding the cycle of bedbugs and the importance of prep. There are sometimes inconsistencies in how that’s communicated and the expectations around that’. (SP1, Social Worker)
Lack of social support	‘They have very limited social support, so there is no one to do the prep for them’. (SP14, Geriatric Psychiatrist)
Poor physical health	‘I have osteoarthritis in both my legs and in my lower back. I’ve had two knee surgeries on my left knee, total knee replacement. I cannot bend down, and I cannot pick things off the floor’. (Tenant 7) ‘If they have a visual impairment, they can’t see the bedbugs. They don’t always react to a bite, and they can’t see the bite’. (SP15, Case Manager)
Memory problems	‘We work with a lot of people with cognitive impairment, and someone will come in and put all the stuff in bags and then the tenant doesn’t remember why [their stuff] is in bags. So, they open them all back up again’. (SP2, Nurse Practitioner)
Lack of financial resources	‘[The housing provider] will come in to treat the unit, but these seniors, they don’t have any income, they can’t pay for any private help to come and help them [prep]’. (SP33, Care Coordinator)
Lack of support services	‘A lot of times, a person can’t get access to [prep] services unless they are going to be evicted’. (SP13, Supportive Housing Manager) ‘The bed bugs end up coming back. [. . .] but it’s hard to find funding for people who get bedbugs repeatedly. Agencies, I find, will fund the first time, then you’re on your own’. (SP31, Care Coordinator)
Excess clutter	‘Hoarding and bedbugs, they go together. Once they have accumulated a lot of things in the apartment, the bedbugs live there’. (SP51, Supportive Housing Nurse)
Fear of repercussions	‘The tenants that are afraid to come forward because they’re afraid of . . . Let’s say, seniors in general, have a feeling where they’re losing some control over their lives. [With bedbugs], the fear is that if someone comes in, maybe they’ll make me throw away all of my belongings, maybe they’ll throw me out, maybe they’ll do something I don’t understand, and I’ll get sick, or whatever’. (Tenant 35)

‘Flawless’ unit preparation included de-cluttering and ‘bag[ging] up all your stuff. Not just your clothes. Everything. Things on the walls, all into bags’ (SP7, Tenant Services Coordinator). This was particularly difficult for tenants living in bachelor apartments ‘where there is nowhere to put all your packed stuff’ (Tenant 35). ‘Living out of bags’ (Tenant 27) was stressful and frustrating, as it was difficult to find belongings among the ‘dozens of bags’ (Tenant 28). Tenants described living in ‘chaos’ (Tenant 25) and noted that preparation process ‘destroyed their life’ (Tenant 21). Throwing out infested furniture was particularly traumatic but commonplace. As one tenant described,

*I had nice drapes on there. I saw one crawling down. Gone. I got rid of my couches. I got rid of my bedding. I washed the clothes so much I don’t think they’re even going to stand up any longer. I can’t afford this anymore. I can’t afford living on the floor, sleeping on the floor, because if I get them again, everything’s going back to the garbage. I’ve just had enough.* (Tenant 39)

Service providers indicated they do ‘the best we can with the hours we have’ (SP13, Supportive Housing Manager) to help with preparation, but most were not resourced to provide full support. There was also a noted gap with the lack of ‘post-prep’ services to help tenants unpack and re-organize their apartment after treatment:

*A big problem [. . .] is there’s not really any post prep. So, you got the prep, you got the treatment, but all your stuff is in bags on the floor now and it’s been three months and your bags are all over the floor and you can’t physically put the stuff away. Now you’re pissed because you agreed to this service, and you didn’t think it was going to disrupt your life and now it has [. . .] and no one is helping you put it away.* (SP6, Social Worker)

### Getting treated – ‘It takes a concentrated effort to help the people that are most vulnerable’

Service providers emphasized the need to work together with tenants, housing staff, and vendors to successfully treat the units. Without effective coordination, service providers described the risks of tenants refusing treatment or bringing infested items back into the newly treated unit because their coats, bags, wheelchairs or walkers, and cat carriers were not steamed:

*You need to organize to have a [personal support worker] there to make sure they get showered and bathed, that they put on clean clothes, and that somebody is there when the pest control guy comes so that he knows he needs to steam down the walker. It’s a lot to coordinate.* (SP7, Tenant Services Coordinator)

One service provider attributed their success to the fact that they were a member of an ‘integrated pest-management table’ that included staff from their agency and the housing provider who worked together to apply a case management approach to tenants with chronic bedbug infestations.

A notable challenge was that ‘people don’t know where to go when they have to be out of the unit for four hours’ (SP7, Tenant Services Coordinator). While one nurse practitioner described how their ‘program has access to a respite unit while they are getting bed bug treatment’ (SP2, Nurse Practitioner), others stressed that tenants ‘don’t have anywhere to go’ (SP24, Care Coordinator) and that it was unreasonable ‘to ask an 80-year-old woman with mobility issues to spend five hours in the lobby’ (SP1, Social Worker). This process was even more complicated for tenants with pets. One tenant discussed the difficulty he had putting his cats in carriers to keep them in the gymnasium all day (Tenant 7), while a service provider described how they ‘put the cat on the balcony and hope it’s okay’ (SP6, Social Worker) because there were no other options. Tenants and service providers alike questioned why there was not a more systematic approach to treatment supports; while tenants described that they were sometimes able to use an empty unit in their building, these were not reliably available.

Quality control with pest vendors was also an issue. Participants were concerned that the housing provider ‘takes the lowest bidder’ (SP52, Supportive Housing Nurse) and had several examples of vendors not coming at the designated day or time, as well as superficially treating the unit. Participants stressed the need to ‘enforce’ proper treatment protocols, as vendors were known to cut corners:

*Sometimes I’m playing the role of, ‘they didn’t get their spraying this week? What happened with that?’ or ‘they’ve had all their sprayings, but it looks like there’s still some bugs there’, you know? Getting them back in. [I have to] advocate, but also [be] the bug inspector as well, it seems.* (SP1)

Tenants wanted to be able to give input on pest control vendors. One tenant exclaimed, ‘we’re the ones that live there, right? If anybody’s going to know, we’re going to know [. . .] we’re going to see [who] is getting results or not getting results’ (Tenant 27). In the absence of effective pest management, tenants took it upon themselves to implement their own measures. They used diatomaceous earth (Tenant 16) and eucalyptus and bleach cleaning solutions (Tenant 17), paid for private fumigation (Tenant 30), and purchased hand-held steamers (Tenant 15 and Tenant 27). Others lobbied their City Councillor for building-wide pest-management initiatives. For instance, one tenant shared a list of 21 written deputations presented at City Council describing their chronic infestations, while another discussed a recent experience organizing a meeting between housing staff and their local councillor:

*We’ve been asking for [a building-wide treatment] for a long time, and we’ve done our own in-house surveys, we’ve followed the pest people around to see how many units they were doing. No one in management was listening to our problems. So, we thought it was time to phone the councillor. It has taken [4 months] to get a response from [housing] to come to a building meeting. [In the end], they said the building is lined up for the full building treatment that we have been asking for since [last year].* (Tenant 35)

### Access to services – ‘There’s a lot of agencies that won’t even go into someone’s place if they have bedbugs’

Tenants experienced bedbug stigma from community services: some were not eligible for services if they had an active infestation, and others were asked not to attend medical appointments until the infestation was cleared:

*Going to physiotherapy, I put my coat on, and I saw that I had bedbugs on my coat that had been super dried twice. I thought, [if I tell them], they’ll make me cancel and they will make me pay for the physio anyways.* (Tenant 15)

Service providers discussed going into infested units only if they felt comfortable. For instance, one nurse practitioner indicated that they would go into units ‘as long as the infestation is not overwhelming, like [if] there’s bedbugs falling off the ceiling, which we have sometimes’ (SP2). In the absence of entering units, service providers would try to meet clients in common areas of the building but cautioned that this was ‘not a practical policy’ (SP1, Social Worker) because many clients are unable to safely leave due to mobility challenges. Other service providers distinguished between providing hands-on care versus psychiatric and mental health support, suggesting that it was more difficult to provide personal care (e.g. bathing) in infested units, especially as the packed bags create safety hazards navigating the unit.

Service providers did not want to ‘be the cause of the bedbugs that spread’ (SP16, CSS Manager). To reduce risk, they implemented a variety of strategies to limit exposure in infested units (see Table 4). Despite the need for these safety measures, some were concerned that they dehumanized their client. For instance, one service provider reflected that ‘when you’re pulling the [full] body suit on, the tenants know there is a real serious problem. Usually the first question is, why am I still here if it’s bad enough for you to put that on?’ (SP8, Tenant Services Coordinator). Another felt it was impossible to maintain a client’s dignity if you bring your own chair into their home (FG34, Care Coordinator).

**Table 4:** Strategies used by service providers to reduce exposure in infested units

Safety strategy	Supporting quote
Wear personal protective equipment	‘It depends on how bad the bedbugs are. So, it can be from just the booties that cover the shoes, if they are not bad at all, to the knee-high booties, to a full suit’. (SP13, Supportive Housing Manager)
Bring a change of clothes	‘I always bring myself a change of clothes or two in my car, because sometimes if I know I’ve been in a unit that is pretty infested, then before I leave the building, I change my clothes’. (SP6, Social Worker)
Bring a stool to sit on	‘Yeah, you can’t really have a conversation with somebody, particularly if you’re getting into complicated stuff, while you’re standing. Plus, I need to be taking notes, like I can’t do that standing up [. . .] if it’s reasonably safe, if I can find like a plastic chair or something to sit on, I’ll do that. But barring all of that, I have a little camp stool just a little foldable camp stool that I take with me, and I just sit on that’. (SP2, Nurse Practitioner)
Education sessions	‘We do extensive training as well with staff. We actually brought [housing’s pest control manager] in one time to give a presentation to staff just around what to do if you’re in an environment with bedbugs. There are techniques and tactics that we teach staff. Some of the things, like you said, the bedbug one, some of the number ones are really like, not using cuffed pants, for example, not sitting, don’t put your things down, checking yourself as soon as you leave. Or leave your belongings in the car or outside the door’. (SP10, CSS Manager)

## Discussion

Our findings shed new light on the experiences of bedbug infestations among low-income older adults and the health and social service providers who support them. Participants described fighting a losing battle against pervasive bedbug infestations. Older tenants faced several obstacles navigating the pest-management process, including difficulties preparing their unit and coordinating treatment. In addition to the detrimental impacts on mental health, bedbugs negatively impacted access to services. Findings point to several opportunities to enhance integrated pest-management strategies to improve outcomes for low-income older adults.

This study highlights the stress that older adults experience managing bedbugs: tenants lived in fear of having to manage another infestation. The process of preparing their unit for treatment felt insurmountable due to physical, mental, social, and financial barriers. For those who were forced to throw away their belongings, the added financial strain of replacing their belongings created additional distress. Many tenants also experienced stigma from service providers who refused services due to bedbugs. While experiences of distress, stigma, and fear have been widely reported in commentaries and case reports over the past decade, very little original research has been conducted.^13^ Therefore, our findings provide some much-needed insights into the concerns of low-income older adults about bedbugs, and how these infestations impact their mental health.

In prior research, spatial factors (e.g. clutter) were recognized as key safety concerns in homecare.^29,30^ Bedbugs, however, were not discussed, despite the potential reluctance to provide care in infested homes.^16^ In this study, service providers discussed the need to balance their personal safety with their duty to clients; however, strategies to reduce exposure in infested units were not always perceived as feasible to implement, particularly when they further stigmatized the client. The lack of best practice guidelines for providing services in infested units also led to inconsistent policies across agencies, creating additional barriers to support.

Older tenants and service providers both expressed concerns over the quality of pest control vendors and the implications this had for the treatment process. Studies show that choosing the lowest cost vendor is not uncommon, but dedicated and careful vendors are needed to ensure success.^8^ As a result of poor-quality vendors, tenants had little trust in their housing provider to manage infestations and resorted to implementing their own initiatives, including mobilizing political ties to advocate for better vendors and building-wide treatment protocols.

### Recommendations

Based on our findings, three recommendations have emerged to enhance the integrated pest-management process for low-income older adults (see Table 5 for a summary).

**Table 5:** Practice recommendations.

Provide More Unit Prep and Unprep Services	More public health funding is needed to support older tenants with unit preparation, furniture replacement, and reorganization (i.e. unprep) after treatment to reduce the physical, psychological, and financial impacts of bedbug infestations.
Include Service Providers as Members of the Integrated Pest-Management Team	Integrated pest-management teams should be expanded to include health and social service providers, as they play a critical role in identifying and reporting infestations, supporting unit preparation, liaising with pest control vendors, and facilitating follow-up preventive measures, including identifying re-emerging infestations, and providing support for factors (e.g. clutter) that place older tenants at risk for re-infestation. Including health and social service providers as members of the integrated pest-management team will allow for the co-creation of best practice guidelines for operating in infested units and facilitate opportunities for training on strategies to identify infestations and reduce personal exposure when supporting clients in infested units.
Enhance Pest-Management Approaches for Older Adults	Social housing providers need to tailor their pest-management approach in response to the unique challenges faced by older tenants. This should include more diligent, building-wide monitoring of infestations, as well as providing resources to support the preparation and treatment process such as mattress encasements, heavy duty garbage bags, and a safe place to stay in during treatment.

### Provide more unit prep and unprep services

Non-compliance with the preparation process is a well-known barrier in pest management.^6–10^ Our findings build on this literature and shed further light on the physical and mental health challenges older tenants face executing and complying with the preparation process. Lack of social support and financial resources exacerbated these issues, and there were very few services available in the community to fill these gaps. There was also a dearth of formal supports to help tenants unpack and re-organize their apartment following treatment. In a noteworthy evaluation of an integrated pest-management approach with low-income older adults,^8^ housing staff were responsible for carrying out preparations. Without resources to support underlying physical, mental, social health challenges, compliance with the preparation requirements is likely to be an ongoing issue.

### Include service providers as members of the integrated pest-management team

Successful integrated pest-management programmes involve a three-way partnership between residents, building managers, and pest managers;^6^ however, our findings identify health and social service providers as key partners. As evidenced by one agency in this study, integrated pest-management meetings between housing staff and service providers allowed for case management approaches to be applied, ensuring that tenants with complex needs were fully supported throughout the treatment process. This practice also facilitated training opportunities for frontline staff on how to identify bedbugs and strategies to reduce exposure in infested units. This type of interprofessional team is further supported by Ashcroft et al.^13^ who called for interdisciplinary collaborations to develop effective strategies to support clients during pest infestations.

### Enhance pest-management approaches for older adults

There are several ways for housing providers to tailor their pest-management approach to better support older tenants. One widely-requested example was a designated unit for older tenants and their pets to use while their unit is being treated. In other studies, housing staff provided mattress encasements and heavy duty garbage bags to older tenants to support unit preparation.^8^

Our findings also highlight the need for more deliberate, building-wide monitoring to address persistent and re-emerging infestations. For instance, Cooper et al.^8^ found that a rigorous bi-weekly post-treatment follow-up schedule with older tenants was important for ensuring infestations were completely cleared. Participants in this study also called for more regular unit inspections as well as more accountability for pest control vendors, as they observed that vendors cut corners during treatment. While this level of monitoring may be more resource intensive,^8^ it may also help re-build trust among tenants that their housing provider is committed to providing a pest-free home.

### Limitations

This study does not capture the experiences of housing administrators and pest managers, who are critical members of the integrated pest-management team. Future research should apply qualitative approaches to consider the barriers and facilitators of integrated pest-management strategies with low-income older adults from these perspectives.

## Conclusion

Older adults in low-income housing communities are more vulnerable to pest infestations and face a variety of obstacles navigating the pest-management process. As a result, many have repeat experiences with bedbugs. There is an urgent need to increase public health funding to support older adults with the cost of bed bug elimination and to enhance pest-management strategies through partnerships with health and social service agencies.
